# Convolutional Neural Networks to Detect Vestibular Schwannomas on Single MRI Slices: A Feasibility Study

**DOI:** 10.3390/cancers14092069

**Published:** 2022-04-20

**Authors:** Carole Koechli, Erwin Vu, Philipp Sager, Lukas Näf, Tim Fischer, Paul M. Putora, Felix Ehret, Christoph Fürweger, Christina Schröder, Robert Förster, Daniel R. Zwahlen, Alexander Muacevic, Paul Windisch

**Affiliations:** 1 Department of Radiation Oncology, Kantonsspital Winterthur, 8401 Winterthur, Switzerland; carole.koechli@uzh.ch (C.K.); philipp.sager@student.unisg.ch (P.S.); christina.schroeder@ksw.ch (C.S.); robert.foerster@ksw.ch (R.F.); daniel.zwahlen@ksw.ch (D.R.Z.); 2 Department of Radiation Oncology, Kantonsspital St. Gallen, 9007 St. Gallen, Switzerland; erwin.vu@kssg.ch (E.V.); paulmartin.putora@kssg.ch (P.M.P.); 3 Department of Radiology, Kantonsspital St. Gallen, 9007 St. Gallen, Switzerland; lukas.naef@kssg.ch (L.N.); tim.fischer@kssg.ch (T.F.); 4 Department of Radiation Oncology, University of Bern, 3010 Bern, Switzerland; 5 Berlin Institute of Health at Charité—Universitätsmedizin Berlin, 10117 Berlin, Germany; felix.ehret@charite.de; 6 Charité—Universitätsmedizin Berlin, Corporate Member of Freie Universität Berlin and Humboldt-Universität zu Berlin, Department of Radiation Oncology, 13353 Berlin, Germany; 7 European Radiosurgery Center, 81377 Munich, Germany; christoph.fuerweger@erc-munich.com (C.F.); alexander.muacevic@erc-munich.com (A.M.); 8 Department of Stereotaxy and Functional Neurosurgery, University of Cologne, Faculty of Medicine and University Hospital Cologne, 50937 Cologne, Germany

**Keywords:** artificial intelligence, deep learning, machine learning, vestibular, schwannoma, neuro-oncology

## Abstract

**Simple Summary:**

Due to the fact that they take inter-slice information into account, 3D- and 2.5D-convolutional neural networks (CNNs) potentially perform better in tumor detection tasks than 2D-CNNs. However, this potential benefit is at the expense of increased computational power and the need for segmentations as an input. Therefore, in this study we aimed to detect vestibular schwannomas (VSs) in individual magnetic resonance imaging (MRI) slices by using a 2D-CNN. We retrained (539 patients) and internally validated (94 patients) a pretrained CNN using contrast-enhanced MRI slices from one institution. Furthermore, we externally validated the CNN using contrast-enhanced MRI slices from another institution. This resulted in an accuracy of 0.949 (95% CI 0.935–0.963) and 0.912 (95% CI 0.866–0.958) for the internal and external validation, respectively. Our findings indicate that 2D-CNNs might be a promising alternative to 2.5-/3D-CNNs for certain tasks thanks to the decreased requirement for computational power and the fact that there is no need for segmentations.

**Abstract:**

In this study. we aimed to detect vestibular schwannomas (VSs) in individual magnetic resonance imaging (MRI) slices by using a 2D-CNN. A pretrained CNN (ResNet-34) was retrained and internally validated using contrast-enhanced T1-weighted (T1c) MRI slices from one institution. In a second step, the model was externally validated using T1c- and T1-weighted (T1) slices from a different institution. As a substitute, bisected slices were used with and without tumors originating from whole transversal slices that contained part of the unilateral VS. The model predictions were assessed based on the categorical accuracy and confusion matrices. A total of 539, 94, and 74 patients were included for training, internal validation, and external T1c validation, respectively. This resulted in an accuracy of 0.949 (95% CI 0.935–0.963) for the internal validation and 0.912 (95% CI 0.866–0.958) for the external T1c validation. We suggest that 2D-CNNs might be a promising alternative to 2.5-/3D-CNNs for certain tasks thanks to the decreased demand for computational power and the fact that there is no need for segmentations. However, further research is needed on the difference between 2D-CNNs and more complex architectures.

## 1. Introduction

Recent advances have allowed for a more widespread application of machine learning (ML) techniques in various fields thanks to improved computational power and user-optimized software libraries [1,2]. The possible applications of ML techniques include automatic tumor detection, segmentation, and classification to diagnose brain tumors based on magnetic resonance imaging (MRI) [3,4]. Accordingly, automatic tumor detection and classification could potentially support radiologists by increasing the efficiency of the clinical workflow and providing a second opinion. Currently, to the best of our knowledge, no commercially available or established implementation can detect or classify different brain tumor entities.

A shift in the investigated ML techniques was reported from traditional and classical ML to deep learning techniques to detect brain tumors based on MRI [5]. Accordingly, 2.5D- and 3D-convolutional neural networks (CNNs) have been implemented in several publications to automatically perform substeps of a tumor diagnosis based on MRI [6,7,8,9,10]. Various 3D-CNNs reportedly performed well in subtasks of a tumor diagnosis using the publicly available Multimodal Brain Tumor Segmentation Challenge (BraTS) data set [8,9,10].

There are distinct benefits and downsides with 3D- and 2D-CNNs. On the one hand, 3D-CNNs take inter-slice information into account, potentially resulting in a superior performance in comparison with 2D-CNNs. On the other hand, 2D-CNNs achieve a higher efficiency in memory and computational power compared with 3D-CNNs [11]. Moreover, many CNNs require segmentations that indicate the region of interest to the algorithm. However, it tends to be less time-consuming to select tumor containing slices as an input for 2D-CNNs than to contour the region of interest as an input for 3D-CNNs [7,12,13]. In addition, data augmentation techniques are better established for 2D-CNNs compared with 3D-CNNs [14,15].

Several techniques have been investigated that automatically segment the regions of interest in MRI slices [16,17]. For example, Kadry et al. used the U-Net scheme to extract ischemic stroke lesions from MRI slices [17].

Various architectures of convolutional neural networks have been investigated for tumor classification and detection including AlexNet, GoogLeNet, and VGGNet [18]. Rehman et al. observed that the highest accuracy was achieved in terms of classification and detection of benign brain tumors using the VGG16 architecture [18].

Vestibular schwannomas (VSs) represent an optimal tumor entity to examine early automatic tumor detection feasibility studies thanks to their relatively constant location compared with most other entities. VSs are located in the cerebellopontine angle and only vary in size, shape, and intratumoral homogeneity [19]. Thus, it is likely that less data are required for training.

Sager et al. investigated the feasibility of VS detection using single MRI slices [20]. MRI was selected as the imaging modality because it is the method of choice to detect VSs in clinical practice [19]. A 2D-CNN was trained and validated without the use of segmentations. However, the study used the laterality of the VS as a surrogate for the tumor detection. A high accuracy was obtained for the prediction of laterality in the internal and external validation cohort, with only a minor reduction in accuracy in the latter. The current study constituted a continuation of the work by Sager et al. using the same data but without the use of laterality as a surrogate [20].

The aim of this work was, therefore, to study the performance of a computationally inexpensive 2D-CNN to detect VSs based on single transversal MRI slices. For this purpose, MRI slices were used to train, internally validate, and externally validate the 2D-CNN for VS detection. Advancements in artificial intelligence were used to investigate the region of interest for the model to detect the VS. Additionally, the 2D-CNN was externally validated using MRI slices without contrast enhancement. This was to test the hypothesis as to whether the model could generalize when applied to the MRI slices without contrast enhancement.

## 2. Materials and Methods

**Figure 1:**
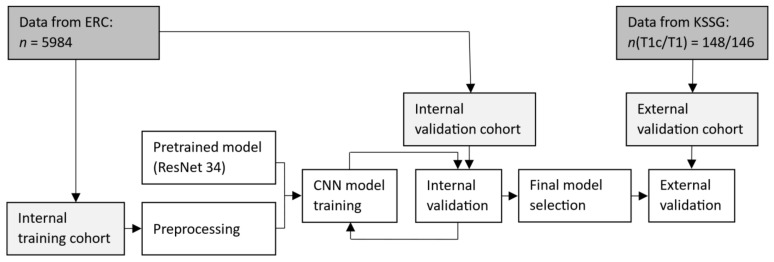
Workflow of the training, internal validation, and external validation. ERC: European Radiosurgery Center in Munich; KSSG: Kantonsspital St. Gallen; *n*: number of slices; T1c: contrast-enhanced T1-weighted; T1: T1-weighted.

A retrospective feasibility study was conducted using the same MRI slices as Sager et al. [20]. The cohorts included an internal training, an internal validation, and an external validation data set. The internal data sets were supplied by the European Radiosurgery Center in Munich (Munich, Germany) whereas the external validation data set was from the Kantonsspital St. Gallen (St. Gallen, Switzerland). The workflow is illustrated in Figure 1.

Overall, the MRI slices were included and excluded as follows: Only patients with a T1-weighted (T1) or contrast-enhanced T1-weighted (T1c) MRI scan of a VS diagnosed by a board-certified radiologist were considered. Hereof, only transversal MRI slices were collected that contained part of the tumor. Accordingly, one or several slices were selected per patient, depending on the tumor size and slice thickness. The internal data were split into training and validation sets before and after a set reference date without a cross-validation. This was to ensure that no slices from the same patient could be present in both the training and validation sets. The MRI slices of patients with bilateral VSs were omitted. Moreover, slices were excluded with a significant tumor spread to the contralateral hemisphere. This elimination step was performed by a radiation oncology resident and a medical student by visually assessing all MRI slices until a consensus was reached. As the internal data set consisted of patients assigned from different clinics in various countries, heterogeneous imaging protocols were used.

For the training and internal validation, T1c MRI slices were included independent of whether or not the patient had received any prior treatment to the tumor before the images had been acquired. Magnetic resonance (MR) images were omitted with artifacts that strongly reduced the overall image quality. 

For the external validation, T1c and T1 MRI slices were consecutively sampled from the radiology database. The MR images were acquired on Siemens devices with field strengths between 1.5 and 3 T and a variable slice thickness ≥ 1 mm. Specifically, T1 MRI slices were included to assess whether the CNN could generalize the slices with an acquisition sequence on which the model had not been trained. Patients with pretreated VSs were excluded from the external validation cohort. This was to prevent the CNN from considering treatment-related modifications when predicting the presence of the VS.

**Figure 2:**
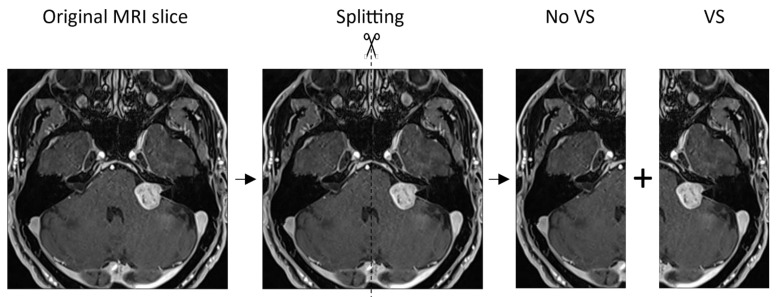
Workflow of splitting the magnetic resonance imaging (MRI) slices into a left and right image, resulting in a hemisphere with and without a tumor.

The MRI slices were preprocessed by labeling and bisecting the images. First, all MR images were labeled from the internal and external cohort to specify whether the VS was located on the left or right side. For the internal data set, the labeling was performed by a radiation oncology resident and a medical student. For the external data set, two board-certified radiologists classified and revised the MRI slices containing the VS, respectively. A Python (version 3.8.6) algorithm was then programmed to automatically split the MRI slices into a left and right image side. By splitting the images, two sliced images were obtained; one hemisphere with and one without a VS, respectively, as illustrated in Figure 2.

The CNN was trained using Python, the fastai library (version 2.4.1), and the PyTorch library (version 1.7.0) [21,22]. The CNN training included the data augmentation and fine-tuning of a pretrained model. All settings were selected according to the precursor publication by Sager et al. to allow the assessment of laterality as a surrogate for tumor detection [20]. First, the data were augmented using RandomResizedCrop and aug_transforms [23]. RandomResizedCrop amplifies data by varying the size and image section [23]. A minimum scale and resample were selected equal to 0.9 and 224 × 224 pixels, respectively. The resampling was chosen as a tradeoff between a fast computational time and maintaining sufficient information. aug_transforms contains a standard set of augmentations including random flipping, zooming, warping, maximum rotation, and maximum change of brightness and contrast (i.e., lighting) [23]. For this purpose, the following settings were defined: no flipping, minimum zooming = 1, maximum zooming = 1.1, no warping, maximum rotation = 15°, and maximum lighting = 0.1. The existing ResNet-34 was selected as a pretrained model, previously trained using the ImageNet data set [24]. It consisted of 34 layers [25]. This specific pretrained model was selected due to its demonstrated ability to classify images [24] as well as the possibility of importing ResNet architectures of varying complexities into different popular deep learning libraries [24]. Finally, the CNN was fine-tuned using a variable learning rate, flattened cross-entropy loss as the loss function, and Adam as the optimizer [26]. For the first five epochs, the body weights of the CNN were frozen and only the head was trained. Subsequently, the body weights were unfrozen and the CNN was trained for another 15 epochs. The training and validation loss were observed to investigate truncation and overfitting.

The performance of the CNN was assessed based on the internal and external data set. For the external validation set, MR images with and without contrast enhancement were separately evaluated. The flattened cross-entropy loss was determined for the 15 unfrozen epochs regarding the training and internal validation cohort. Furthermore, the following performance metrics were calculated for the internal, external T1c, and external T1 validation cohort: the accuracy and corresponding 95% confidence interval (CI); the sensitivity; the specificity; and the F1 score. The 95% CI was computed as follows: CI = 1.96∗sqrt((accuracy∗(1-accuracy))/*n*) with *n* = size of the corresponding validation cohort. Additionally, the confusion matrices were plotted for the external validation cohort with and without contrast enhancement, respectively. Moreover, all external T1c and T1 slices were sorted and displayed, respectively, based on whether they were classified correctly or incorrectly. Finally, the gradient-weighted class activation mapping (Grad-CAM) images were evaluated for five correctly and five incorrectly classified sample images [27]. In doing so, the focus of the CNN was determined during the prediction-making process. Supplementary Material S1 includes the programming code used to split the MRI slices and train, validate, and assess the network.

A literature search was further conducted to compare the findings of this study with previous publications. For this purpose, the Medical Literature Analysis and Retrieval System Online (MEDLINE) database was searched on 31 January 2022 using the PubMed interface. The query used was taken from the precursor publication by Sager et al.: “((vestibular schwannoma[Title]) OR (vestibular schwannomas[Title])) AND ((deep[Title]) OR (network[Title]) OR (networks[Title]) OR (artificial intelligence[Title]) OR (machine learning[Title]))” [20].

Written informed consent was received from all patients to analyze the anonymized clinical and imaging data. Institutional policies were followed when working with the imaging data. For the internal cohort, approval was granted by the institutional review board of the Ludwig Maximilian University of Munich (project 20–437, 22 June 2020) as part of a larger project. This publication is a side project of a larger project modelling the outcomes after radiosurgery. For the external validation cohort, the Ethics Committee of Eastern Switzerland (EKOS 21/041, 9 March 2021) waived the ethical approval, stating that single MRI slices preserve adequate anonymization.

## 3. Results

The characteristics of the data sets are summarized in Table 1, including the number of patients and corresponding MRI slices as well as the VS location. In the training and internal validation cohort, 18 patients (2.8%) previously received radiotherapy and 112 patients (17.7%) formerly underwent a (partial) resection of the VS or suffered a relapse. In the external validation cohort, a few patients did not have both a T1 and a T1c image available. Therefore, the external validation cohort included 82 patients in total (65 patients with a T1 and T1c MR image, eight patients with a T1 MR image only, and nine patients with a T1c MR image only). Overall, no MRI slices were identified with a significant crossing to the contralateral image side. Only two MRI slices were identified in the training, external T1c, and external T1 data set, respectively, with an insignificant intersection.

**Figure 3:**
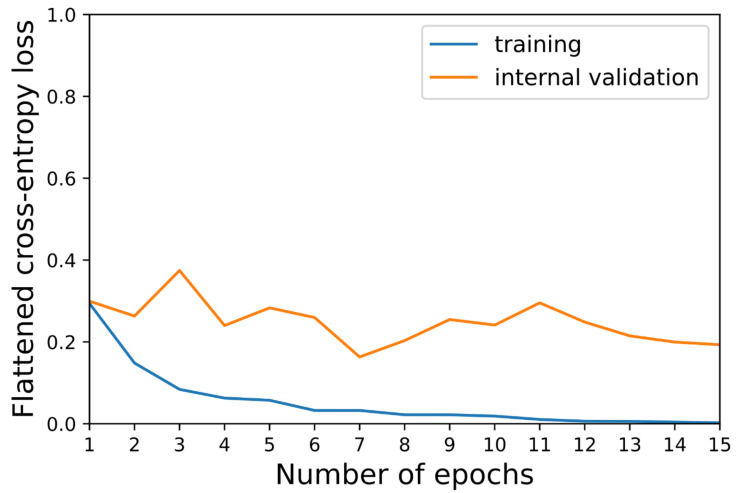
Flattened cross-entropy loss of the training (blue) and internal validation (orange) data set across the 15 unfrozen epochs.

**Figure 4:**
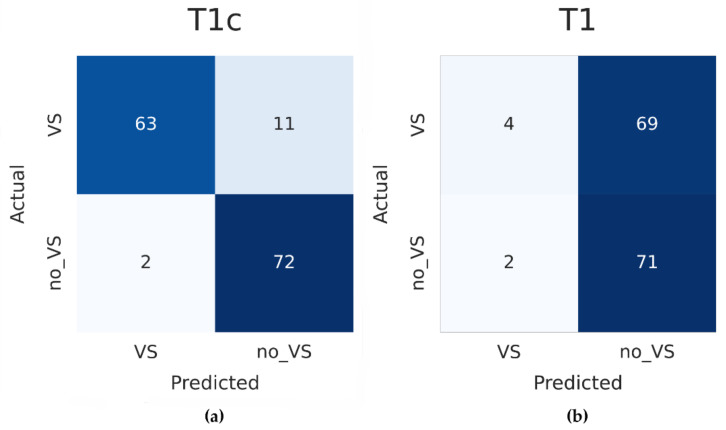
(**a**) Confusion matrix of the external validation with T1c MRI slices; (**b**) confusion matrix of the external validation with T1 MRI slices. VS: vestibular schwannoma.

**Figure 5:**
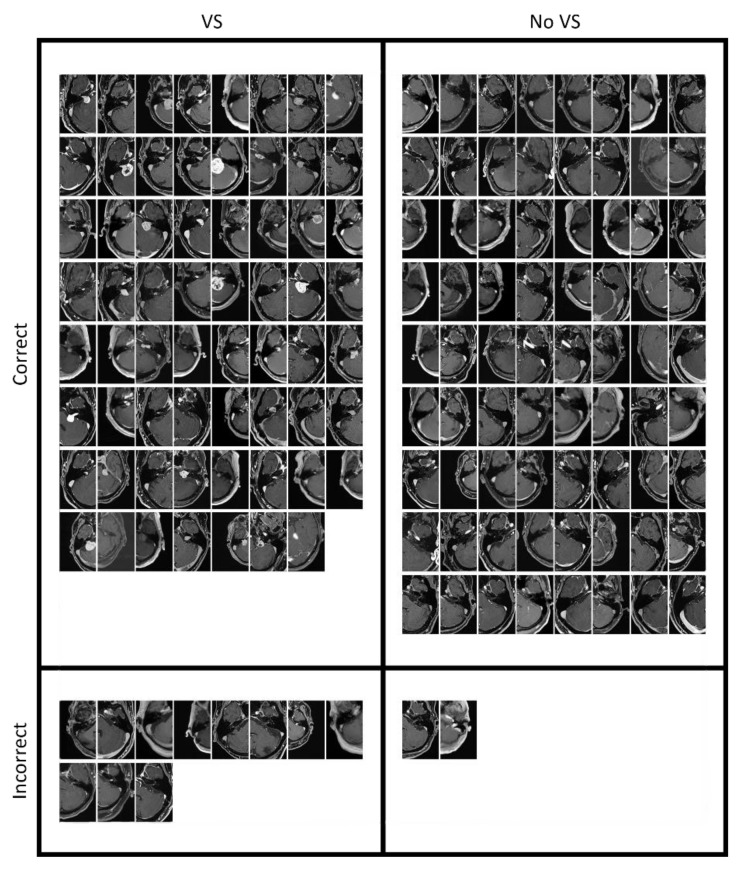
All MRI slices of the external validation data set with the T1c sequence. The images are sorted based on whether they contained a VS and whether they were correctly classified.

**Figure 6:**
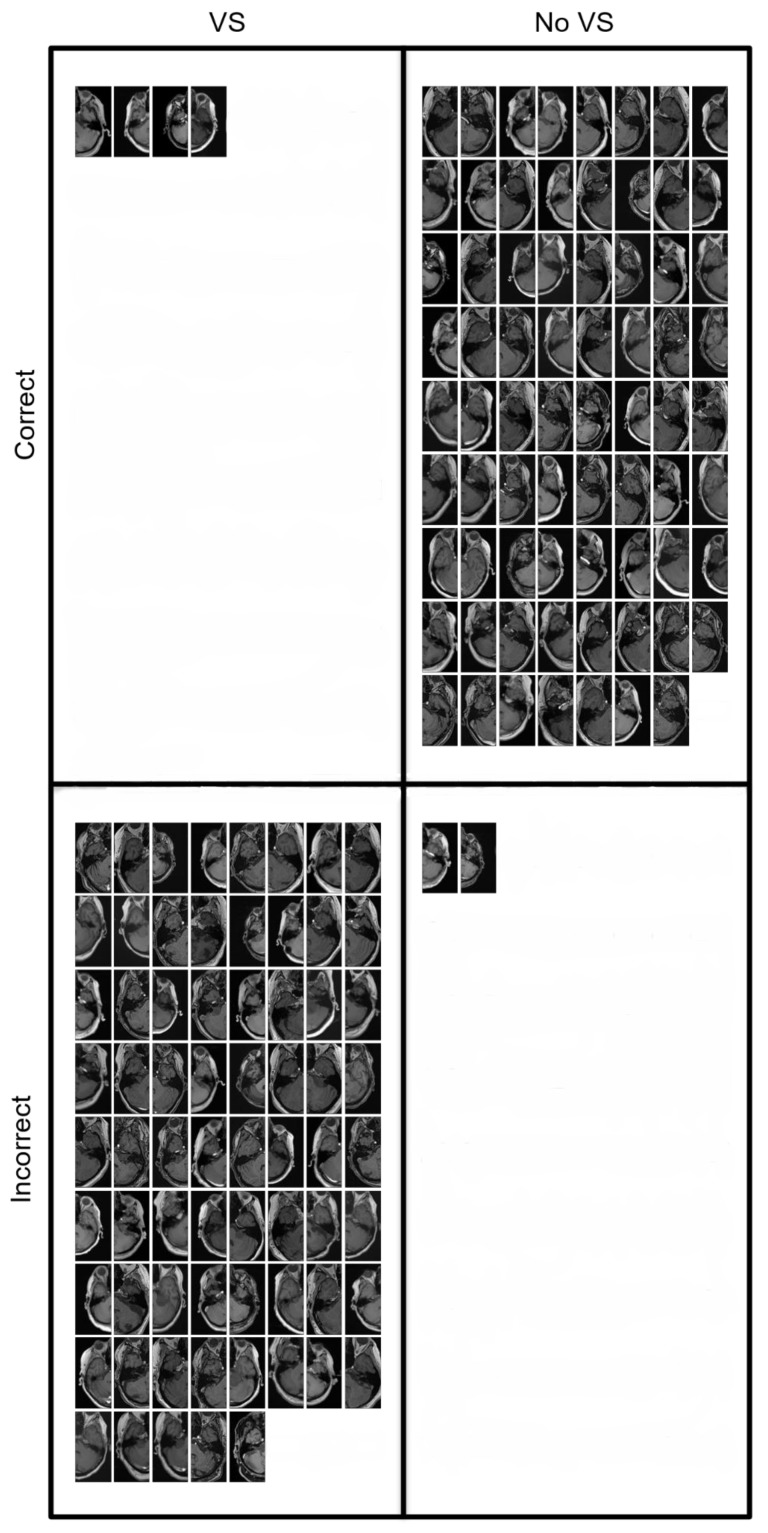
All MRI slices of the external validation data set with the T1 sequence. The images are sorted based on whether they contained a VS and whether they were correctly classified.

The flattened cross-entropy loss is depicted in Figure 3 for the training and internal validation. Table 2 summarizes the performance metrics of the internal, external T1c, and external T1 validation. The confusion matrix is illustrated in Figure 4 for the external validation using the T1c images and T1 images, respectively. The slices are shown in Figure 5 and Figure 6 for the external T1c and T1 validations. The slices were grouped based on whether they were correctly or incorrectly classified by the CNN. They included VSs of varying sizes and shapes as well as blood vessels in the cerebellopontine angle.

**Figure 7:**
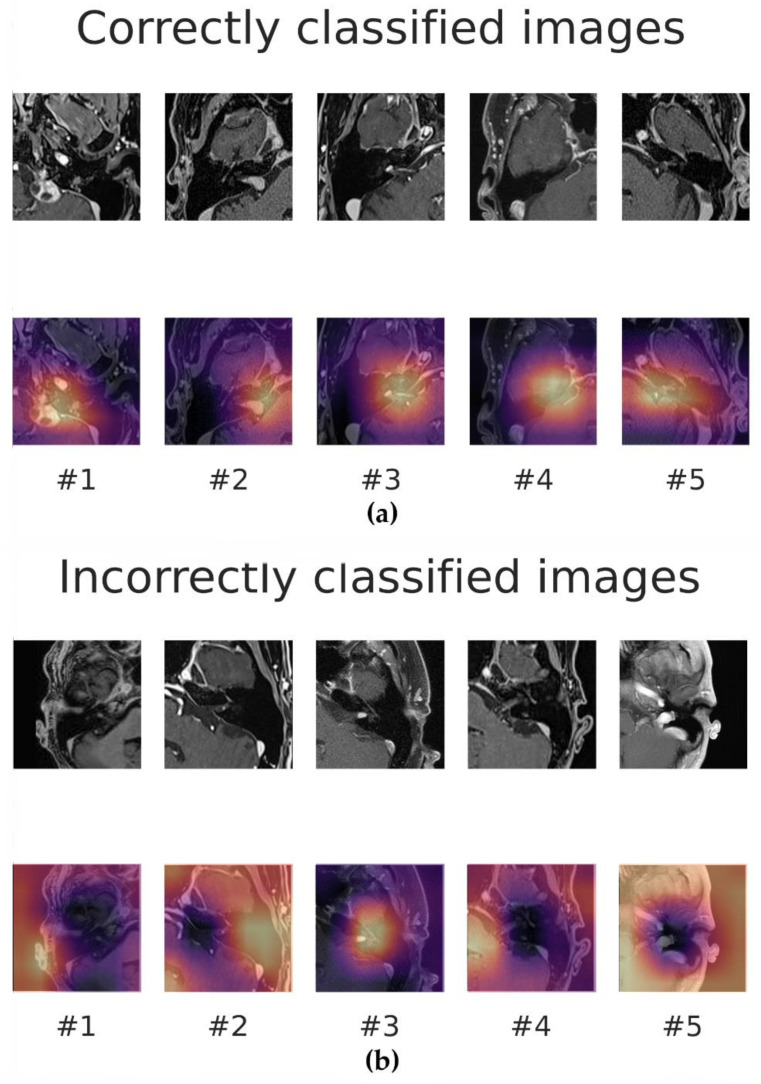
Sample MRI slices of the T1c data set with and without gradient-weighted class activation mapping (Grad-CAM). (**a**) The correctly classified images are shown above; (**b**) the incorrectly classified slices are shown below. Bright yellow and purple correspond with high and low activation, respectively.

Figure 7 shows five correctly and incorrectly classified examples of Grad-CAM images and the corresponding MRI slices for the external T1c validation, respectively. The CNN seemed to focus on a larger area surrounding the tumor including the cerebellopontine angle for the correctly classified slices (slices #1–#5). In contrast, the model mostly concentrated on a larger image section (slices #1, #2, #4, and #5) in the case of the incorrectly classified images. However, in the case of one incorrectly classified image (slice #3), the model focused on the cerebellopontine angle without detecting the VS.

The literature search resulted in 18 publications; no paper reported on the detection of VSs apart from the previously mentioned study by Sager et al. [20,28,29,30,31,32,33,34,35,36,37,38,39,40,41,42,43,44]. Out of all the search results, five publications investigated the automatic segmentation and volumetry of VSs [28,29,30,31,32]. This was, amongst others, to detect tumor growth for clinical management and to evaluate the treatment response after radiosurgery [28,29,30,31,32]. Seven papers examined the clinical factors of VSs including the prediction of postoperative complications, prolonged length of stay after resection, hearing preservation, recurrence, decision-making on treatment, and blood supply to the VS [33,34,35,36,37,38,39]. One paper focused on the segmentation and cystic components of a VS as well as the tumor response after radiosurgery [40]. Three reports explored the genetic aspects including gene expression, disease-related genes, pathways, and potential therapeutic drugs [41,42,43]. One publication reviewed the literature regarding the prophylaxis of deep venous thromboses [44].

## 4. Discussion

Overall, a high accuracy was achieved for both the internal and external validation using contrast enhancement, with only a minor reduction in accuracy in the latter. When comparing these results with those of Sager et al., the performance appeared to be fairly similar [20]. An accuracy of 0.949 (95% CI 0.935–0.963) was obtained for the internal validation in our work compared with 0.974 (95% CI: 0.960–0.988) in the previous study. Likewise, an accuracy of 0.912 (95% CI 0.866–0.958) was obtained for the external T1c validation in the present study in comparison with 0.928 (95% CI: 0.869–0.987) in Sager et al. [20].

In contrast, the CNN could not generalize the slices without contrast enhancement, which was contrary to the previous study. The accuracy was 0.514 (95% CI 0.433–0.595) in this study versus 0.795 (95% CI: 0.702–0.888) in Sager et al. [20]. We hypothesized that the network compared the anatomy of the two hemispheres, if available, to locate the tumor when no contrast enhancement was used. However, no comparison could be made in the case of split slices and the performance of the network dropped to being almost random. The performance of the model could be further analyzed when trained with non-contrast-enhanced slices.

These findings illustrated that, on one hand, laterality was an appropriate surrogate for tumor detection based on T1c slices. One the other hand, there were pitfalls to this approach; e.g., when using slices without contrast enhancement, which led to a significant increase in the prediction performance of the surrogate compared with the actual task.

There are many possible reasons for the high accuracy of our results regarding the contrast-enhanced slices of the external validation data. Extensive data augmentation was implemented for the training of the CNN. Moreover, the training data set contained highly heterogeneous MRI studies.

Only a few MRI slices were classified incorrectly regarding the contrast-enhanced external validation. The misclassified images mostly included slices with fairly small tumor portions or blood vessels in the cerebellopontine angle. Notably, the CNN tended to show more false-negative results (eleven slices) than false-positive results (two slices), as seen in the corresponding confusion matrix. Today, many MRIs use relatively thin slices. Consequently, most MRI scans include at least one slice with a larger tumor portion. Therefore, the CNN could be expected to detect the VS in the vast majority of cases.

Grad-CAM can be used to explain the decision-making process of ML models [27]. Thereby, a more widespread application of ML is supported in general but also in particular in medicine by removing its reputation as a black box [45]. Additionally, Grad-CAM can support the development of ML models [46]. Grad-CAM was implemented in this work to identify the region of interest to detect the VS. Additionally, Grad-CAM observations led to the hypothesis that a CNN could be applied to MRI slices without contrast enhancement by Sager et al. [20].

This study had several limitations. Firstly, split MRI slices were used to assess the feasibility of automatic tumor detection rather than entire MRI slices. However, this allowed a direct comparison with the results of Sager et al. [20]. Additionally, fewer MRI slices were needed to train and validate the model to assess the feasibility of the VS detection. Secondly, MR images were included of pretreated patients for training and internal validation. Therefore, a smaller amount of training data were required at the expense of consistency between the cohorts used for training and internal validation versus external validation. Thirdly, the study comprised a qualitative assessment of Grad-CAM to investigate the region of interest of the CNN whilst lacking a quantitative assessment. Further investigations are needed to determine the optimal hyperparameter settings for training. 

This study had several strengths. The CNN was trained using highly inhomogeneous data including MRI scans from internationally assigned patients and the validation cohorts contained studies from different scanners with different field strengths and slice thicknesses. Furthermore, the training data were augmented using various techniques and the CNN was internally and externally validated with a sizable number of patients. Thereby, the ability of the model to generalize the data from other institutions was assessed. This is, unfortunately, not frequently demonstrated in medical ML research [47].

Automatic tumor detection can potentially increase the efficiency of the clinical workflow by supporting radiologists. It can also provide a second opinion on the existence of a tumor. VS detection is of major significance to offer adequate treatment including surgery, fractionated radiotherapy, radiosurgery, and observations [19]. For example, single fraction stereotactic radiosurgery represents a highly effective treatment with little treatment-related toxicity for small to middle-sized VSs [48].

To build on the findings of this study, the performance of the network on whole MRI slices could be tested in a cohort that also contains bilateral VSs. Moreover, it could be investigated whether training on whole slices with and without VSs results in a different performance compared with using split images. Additionally, a model could be established that combines several VS predictions based on single slices from one patient into one final VS prediction. This combination would constitute a computationally inexpensive way to benefit from an inter-slice context without needing a more powerful graphics processing unit (GPU). Finally, a comparison between this model and a 3D-CNN could analyze the performance of the two models. The research results indicate that 3D-CNNs may perform better for certain tasks compared with 2D-CNNs [49].

## 5. Conclusions

This work demonstrated that it was feasible to detect VSs in split contrast-enhanced MRI slices without the need for segmentations. An accuracy of 0.912 was obtained for the external contrast-enhanced validation data set by using the mentioned training procedure and data; therefore, 2D-CNNs may constitute a promising option for tumor detection thanks to a potentially increased efficiency in data labeling and model training and no need for segmentations. However, further investigations are required on the comparison of 2D-CNNs with more complex architectures, in particular for more challenging research questions. Moreover, it needs to be examined whether split slices constitute an adequate replacement for tumor detection based on unmodified MRI slices.

## Figures and Tables

**Table 1:** Characteristics of the included patients and the corresponding magnetic resonance slices (MRI) slices. T1c: contrast-enhanced T1-weighted; T1: T1-weighted.

Characteristics	Training (T1c)	Internal Validation (T1c)	External Validation (T1c)	External Validation (T1)
Number of patients	539	94	74	73
Number of MRI slices/bisected MRI slices	2538/5076	454/908	74/148	73/146
Tumor location				
Left (number of patients/MRI slices/bisected MRI slices)	278/1307/2614	54/270/540	31/31/62	34/39/78
Right (number of patients/MRI slices/bisected MRI slices)	261/1231/2462	40/184/368	43/43/86	39/39/78

**Table 2:** Performance metrics of the internal, external T1c, and external T1 validation cohort. 95% CI: 95% confidence interval.

Data Set	Accuracy (95% CI)	Sensitivity	Specificity	F1 Score
Internal validation	0.949 (95% CI 0.935–0.963)	0.916	0.982	0.948
External T1c validation	0.912 (95% CI 0.866–0.958)	0.851	0.973	0.906
External T1 validation	0.514 (95% CI 0.433–0.595)	0.055	0.973	0.101

## Data Availability

The corresponding author will provide the data presented in this study upon reasonable request. In Figure 5 and Figure 6, we provided the data used to externally validate the CNN.

## Supplementary Materials

The following supporting information can be downloaded at: https://www.mdpi.com/article/10.3390/cancers14092069/s1, Supplementary Material S1: The programming code used to split the MRI slices and train, validate, and assess the network.
